# Mediating effects of physical activity, BMI, and dietary iron intake on the relationship between depression and chronic headaches

**DOI:** 10.1038/s41598-025-31993-0

**Published:** 2025-12-18

**Authors:** Fahimeh Alsadat Hosseini, Samaneh Bagherian, Maryam Shaygan, Cristina Cañete-Massé, Mitra Bonyani, Farid Najafi

**Affiliations:** 1 Community Based Psychiatric Care Research Center, School of Nursing and Midwifery, Shiraz University of Medical Sciences, Shiraz, Iran; 2 Department of Operating Room, School of Paramedical Sciences, Geriatric Health Research Center, Birjand University of Medical Sciences, Birjand, Iran; 3 Department of Social Psychology and Quantitative Psychology, Faculty of Psychology, Universitat de Barcelona, Barcelona, Spain; 4 Medical Education Development Center, Kermanshah University of Medical Sciences, Kermanshah, Iran; 5 Department of Nutrition Sciences, Research Center for Environmental Determinants of Health (RCEDH), Health Institute, Kermanshah University of Medical Sciences, Kermanshah, Iran

**Keywords:** Depression, Chronic headache, Body mass index, Physical activity, Dietary iron, Mediation analysis, Human behaviour, Risk factors, Depression, Chronic pain

## Abstract

Depression is a significant psychiatric condition linked to chronic headache outcomes, necessitating an exploration of the mechanisms involved in this relationship. This study aimed to examine whether physical activity, body mass index (BMI), and dietary iron intake mediate the relationship between depression and chronic headaches using path analysis. This study employed a cross-sectional approach. A total of 9,918 participants aged 35–65 years were selected through convenience sampling from the baseline phase of the Ravansar Non-Communicable Disease (RaNCD) Cohort Study, part of the larger PERSIAN cohort. Data collection included sociodemographic and clinical information, alongside standardized self-report instruments for assessing physical activity and dietary intake (Food Frequency Questionnaire). The statistical analyses were performed using SPSS version 22 and MPLUS version 8.3. Path analysis was used to assess the direct, indirect, and total effects of depression on chronic headaches. The results demonstrated a significant direct effect of depression on chronic headaches (β = 0.07, 95% CI: 0.05 to 0.08). Additionally, significant indirect effects were observed through the mediating variables of BMI (β = 0.001, 95% CI: 0.000 to 0.002) and dietary iron intake (β = 0.002, 95% CI: 0.001 to 0.002). In contrast, the indirect pathway through physical activity was not statistically significant (β = 0.000, 95% CI: -0.001 to 0.000). Increased BMI and reduced dietary iron intake, influenced by depression, were associated with a higher prevalence of chronic headaches. These findings suggest that interventions targeting chronic headaches should not only address depressive symptoms but also incorporate strategies for weight management and improving dietary iron intake. A comprehensive approach that integrates both psychological and physiological components may enhance treatment outcomes. Future studies employing longitudinal designs are warranted to further elucidate these relationships and inform more effective intervention strategies.

**Supplementary Information:**

The online version contains supplementary material available at 10.1038/s41598-025-31993-0.

## Introduction

Depression is a prevalent and disabling disorder characterized by anhedonia, low self-worth, cognitive impairments, sleep disturbances, and suicidal ideation. As a leading global cause of disability, it negatively affects educational, occupational, and social functioning, as well as overall quality of life^1–3^. Worldwide prevalence has been reported to reach up to 33%, with particularly high rates in the Middle East and South Asia^2^; in Iran, depression accounts for approximately 35–45% of all mental health disorders^3^.

Given the high burden of chronic daily headaches^4^ and the recognized contribution of depression, it is crucial to understand the mechanisms linking these conditions. The biopsychosocial model conceptualizes chronic pain, including headaches, as the result of interactions among biological, psychological, and social factors^5^. Neurophysiological processes, emotional distress, lifestyle behaviors, and social context collectively influence headache onset and persistence^6^. Key modifiable factors—dietary iron intake, physical activity, and body mass index (BMI)—have been associated with both depression and chronic headaches, and evidence suggests that depression may disrupt iron metabolism and utilization^7,8^.

Depression has been associated with disrupted iron homeostasis, including altered absorption, increased excretion, and dysregulation of hormones such as hepcidin and ferroportin^9^. Individuals with depression also tend to consume less dietary iron, despite similar use of supplements^10^. Low iron intake, in turn, is linked to a higher risk of chronic headaches^11,12^; for example, Meng et al. (2021) found an inverse relationship between dietary iron and severe headache prevalence, particularly among women aged 20–50^11^. Insufficient iron intake may also reduce physical activity, which is inversely related to both depression and chronic headaches^13^.

Previous studies have consistently reported a negative association between depression and physical activity^14–16^. For example, Belvederi Murri et al. (2020)^15^, Kaseva et al. (2019)^14^, and Rutherford et al. (2022)^16^ found that individuals with depression are less likely to engage in physical activity. Reduced activity is also linked to higher headache prevalence^17,18^ and may contribute to weight gain and elevated body mass index (BMI)^19^, both associated with depression and chronic headaches.

Studies have shown that depression is linked to greater abdominal adiposity and higher overall BMI^20,21^. Longitudinal data also suggest that major depression during adolescence predicts elevated BMI in adulthood^22^. Elevated BMI, in turn, increases the risk of chronic headaches. Kristoffersen et al. (2020) reported higher headache risk in individuals with BMI over 25^23^, and a meta-analysis by Martami et al. (2022) confirmed a nonlinear relationship between BMI and migraine prevalence, with obese individuals at significantly higher risk (OR = 1.28, 95% CI: 1.15–1.43)^24^.

Previous studies have linked depression with physical activity^14–16^, BMI^25,26^, and dietary iron intake^10,27^, all of which are also associated with chronic headaches^11,18,28^. However, it remains unclear whether physical activity, BMI, and dietary iron intake mediate the relationship between depression and chronic headaches. This study addresses this gap within a biopsychosocial framework, providing new insights into the mechanisms through which depression may contribute to chronic headaches. We hypothesized that physical activity, BMI, and dietary iron intake jointly mediate this association.

## Materials and methods

### Study design and participants

This cross-sectional study, which included a mediation analysis, utilized data from the baseline phase of the Ravansar Non-Communicable Disease (RaNCD) cohort, a component of the larger Prospective Epidemiological Research in Iran (PERSIAN) project. Ravansar is located in Kermanshah Province in western Iran, near the border with Iraq. It encompasses both urban and rural regions, with an estimated Kurdish population of approximately 50,000. The RaNCD cohort began in November 2014 and remains ongoing. For the present analysis, data from the recruitment phase were used. Additional details are available in the published cohort protocol^29–31^.

Participants included in this study were individuals aged 35 to 65 years who took part in the baseline phase of the RaNCD cohort. The study applied several exclusion criteria, including unwillingness to participate; residence in Ravansar for less than nine months per year; being a new resident (less than one year); and inability to attend the cohort center or communicate with interviewers due to physical disabilities, acute psychological conditions, or sensory impairments such as blindness, deafness, or muteness^31^. Furthermore, participants with incomplete data for the primary variables of interest were excluded from the analysis (*n* = 130). The final analytic sample consisted of 9,918 individuals.

### Data collection

Eligible participants received detailed information about the objectives and procedures of the study from a trained professional. Those who agreed to participate signed informed consent forms and were subsequently invited to the study center. Each participant then took part in a private, face-to-face interview, conducted in accordance with the standardized protocols of the cohort study. The collected data were reviewed on the same day by the center supervisor to ensure accuracy and completeness before being electronically recorded. To address any issues or inconsistencies, a follow-up system was implemented whereby participants could be re-invited to the center for further clarification or to complete additional questionnaires, if necessary. Standardized electronic questionnaires were used in line with the PERSIAN cohort protocol. These instruments underwent a thorough revision process after an initial pilot phase to enhance their validity and reliability^30,31^.

### Measures

In the present study, data were collected on demographic and clinical characteristics, including depression and chronic headaches. In addition, selected variables such as physical activity, BMI, and dietary iron intake were assessed.

#### Sociodemographic and clinical characteristics

Sociodemographic and clinical data—including age, gender, educational attainment, marital status, and medical history—were collected using electronic questionnaires administered by trained interviewers. The digital survey also included items related to personal behaviors, such as levels of physical activity. Detailed descriptions of the study’s design and rationale have been previously published and are available for reference^30,31^.

#### Physical activity assessment

Participants’ physical activity was assessed using a validated physical activity questionnaire developed as part of the PERSIAN cohort study. This instrument consisted of 22 items addressing participants’ daily physical activities. Based on their reported activity levels, participants were categorized according to weekly metabolic equivalent task (MET) hours using predefined cutoff points. Specifically, individuals reporting 24 to 36.5 MET-hours per week were classified as having low physical activity; those reporting 36.6 to 44.9 MET-hours per week were considered moderately active; and those with 45 or more MET-hours per week were classified as having high physical activity, as specified in the cohort protocol^31^.

#### BMI measurement

During the recruitment phase of the RaNCD cohort study, participants’ height and weight were measured. BMI was then calculated by dividing weight (in kilograms) by the square of height (in meters)^31^. Body composition, including weight and BMI, was assessed using the InBody 770 device (InBody Co., Seoul, Korea), which provides a weight measurement accuracy of ± 0.5 kg.

#### Dietary iron intake assessment

Dietary intake was evaluated using a validated semi-quantitative food frequency questionnaire (FFQ)^32,33^. In this study, the RaNCD FFQ questionnaire captured information on 31 nutritional components derived from 45 food items. These components included vitamin A, vitamin B6, vitamin B12, vitamin C, vitamin D, vitamin E, folic acid, niacin, iron, zinc, selenium, magnesium, beta-carotene, caffeine, thiamin, riboflavin, onion, garlic, tea, omega-3 and omega-6 fatty acids, trans fats, saturated fatty acids (SFAs), cholesterol, monounsaturated fatty acids (MUFAs), polyunsaturated fatty acids (PUFAs), fiber, protein, total fat, carbohydrates, and total energy intake.

For the purpose of this study, dietary iron intake was specifically analyzed. Iron intake was estimated by multiplying the reported consumption frequency and standard portion size of iron-rich food items by their corresponding iron content, as provided by the Iranian Food Composition Table. Major sources of dietary iron in the FFQ included red meat, liver, legumes (e.g., lentils and beans), leafy green vegetables (e.g., spinach), eggs, and iron-fortified bread and cereals. The total daily iron intake was computed by summing the estimated iron content across all relevant food items.

#### Depression assessment

The presence of depression, whether symptomatic or asymptomatic, was determined either through clinical evaluation by a psychologist or based on self-reported use of antidepressant medications.

#### Chronic headache assessment

The primary outcome of this study was chronic headache, defined as experiencing headaches on 15 or more days per month for a duration of at least three consecutive months^34^. Particular attention was also paid to probable medication-overuse headache, a subtype of chronic daily headache. Medication-overuse headache is considered a secondary condition that arises from the excessive use of acute headache medications in individuals with a predisposition to primary headache disorders^35^.

### Statistical analyses

From the initial sample of 10,048 participants, 130 cases (1.3%) were excluded due to missing data on key study variables. The number of missing cases per variable was as follows: depression (*n* = 42), physical activity (*n* = 35), BMI (*n* = 18), dietary iron intake (*n* = 25), and headache frequency (*n* = 10). Given the low proportion of missing data, listwise deletion was applied and deemed appropriate, with minimal risk of introducing bias. As a result, the final analyses included 9,918 participants.

**Fig. 1:**
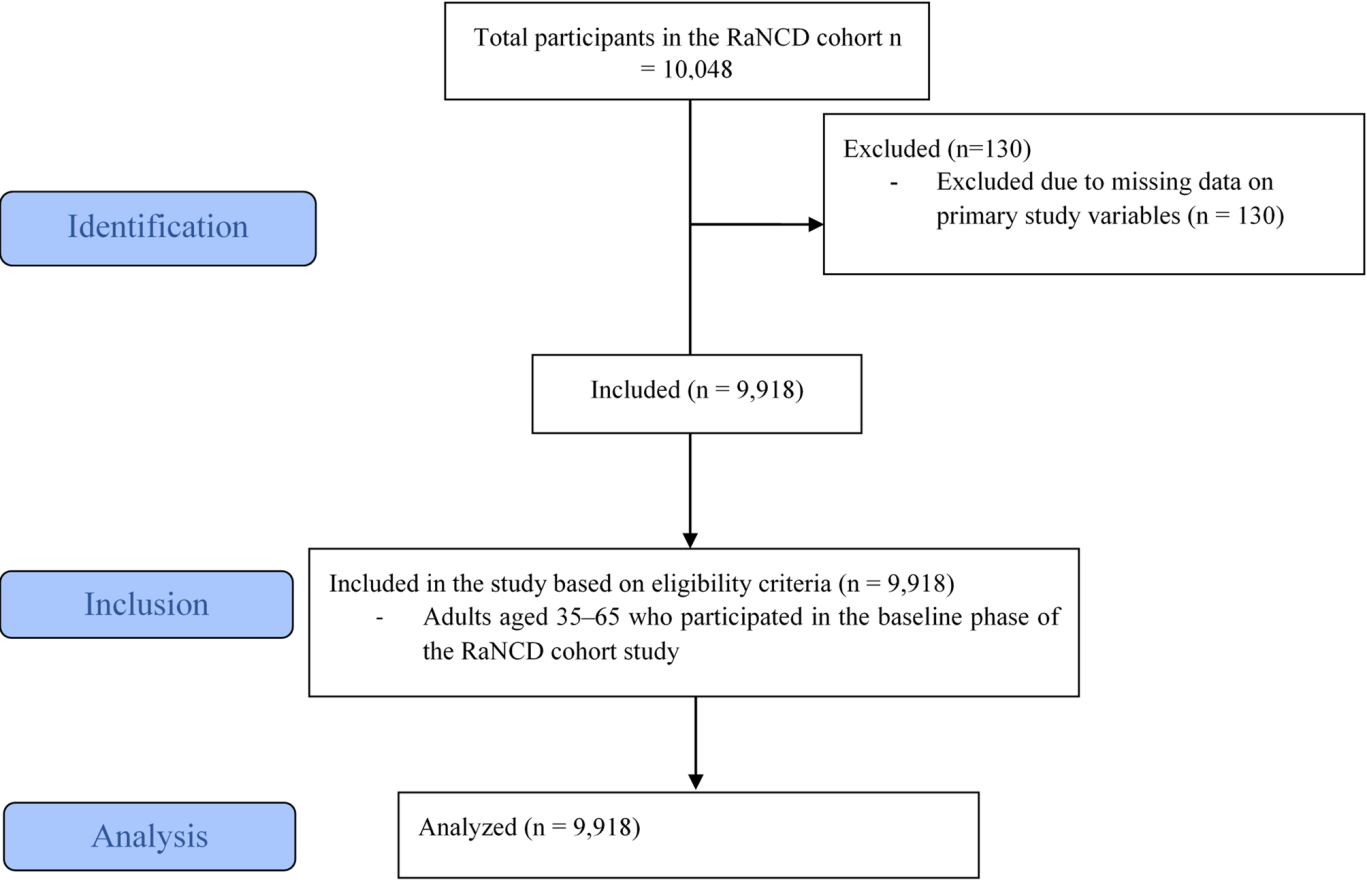
STROBE flow chart.

A STROBE (Strengthening the Reporting of Observational Studies in Epidemiology) flow diagram illustrating the study selection process is presented in Fig. 1. Additionally, the completed STROBE checklist is provided as a supplementary file.

Descriptive statistics, including means, standard deviations, and frequencies, were computed to summarize participants’ sociodemographic characteristics. Differences between individuals with and without depression were examined using independent-samples *t*-tests.

Prior to the main analyses, statistical assumptions were evaluated. Data normality was assessed using skewness and kurtosis, with values between − 2 and + 2 for skewness and − 7 and + 7 for kurtosis indicating an acceptable distribution^36^. Outliers were identified based on standardized scores exceeding 3.29, consistent with established thresholds for extreme observations. Multicollinearity among predictor variables was examined through Pearson’s correlation coefficients and variance inflation factors (VIFs)^37^.

To identify potential confounders, bivariate correlations were conducted between demographic and study variables. Demographic factors showing significant associations (*p* < 0.05) with independent, mediator, or dependent variables were included as covariates in the mediation analyses.

Path analysis, estimated using the maximum likelihood method, was performed to assess the direct and indirect effects of depression on chronic headaches, mediated by physical activity, BMI, and dietary iron intake. Indirect (mediated) effects were tested through bootstrapping with 5,000 resamples, and were considered significant when the 95% bias-corrected confidence intervals did not include zero^38^.

Model fit was examined using several indices, including the normed chi-square (χ²/df), Comparative Fit Index (CFI), Tucker-Lewis Index (TLI), Root Mean Square Error of Approximation (RMSEA), and Standardized Root Mean Square Residual (SRMR). Fit was considered acceptable when CFI and TLI exceeded 0.90, RMSEA was below 0.05, and SRMR was below 0.08^39^. All analyses were conducted in Mplus version 8.3.

The robustness of the model was further tested through sensitivity analyses. All potential confounders were controlled for, and alternative models were evaluated by examining the separate and combined effects of physical activity, body mass index (BMI), and dietary iron intake.

### Ethical considerations

This study was conducted in full accordance with the principles outlined in the Declaration of Helsinki. Ethical approval was obtained from the Ethics Committee of Kermanshah University of Medical Sciences (Approval Code: IR.KUMS.REC.1403.730). Participation in the study was entirely voluntary. All participants were informed about the study objectives and provided written informed consent prior to enrollment. They were assured of their right to withdraw from the study at any time without consequence, and participant anonymity and confidentiality were strictly maintained throughout the research process.

## Results

### Participants

The study included a total of 9,918 individuals aged between 35 and 65 years, selected through convenience sampling from the baseline phase of the cohort study. The mean age of participants was 47.32 ± 8.27 years. The majority were female (52.70%), married (90.28%), and had no formal education (45.83%) (Table 1). Most participants did not report symptoms of depression (96.82%) or chronic headaches (89.19%).

The average level of physical activity was 41.01 ± 8.20 MET-hours per week, indicating a moderate level of activity. The mean BMI was 27.51 ± 4.64 kg/m², and the mean daily dietary iron intake was 19.64 ± 8.10 milligrams.

Independent samples t-tests indicated significant differences between depressed and non-depressed participants in terms of age (*p* = 0.015), BMI (*p* < 0.001), physical activity (*p* < 0.001), and presence of chronic headaches (*p* < 0.001). However, no significant differences were observed for marital status (*p* = 0.71) or educational attainment (*p* = 0.46).

**Table 1:** Sociodemographic profile of the study participants (*n* = 9918).

Variables	Female	Male	Total
**Age** (year) M (SD)*	47.61 (8.45)	47.00 (8.05)	47.32 (8.27)
**Marital status** N (%)** **Single** **Married** **Widowed** **Divorced**	321 (6.14) 4395 (84.08) 422 (8.08) 89 (1.70)	92 (1.96) 4559 (97.19) 7 (0.15) 33 (0.70)	413 (4.16) 8954 (90.28) 429 (4.32) 122 (1.24)
**Educational level** N (%) **Illiterate** **Elementary school** **Middle school** **High school diploma** **Associate degree** **Bachelor’s degree** **Master’s degree** **PhD. degree**	3273 (62.62) 1280 (24.49) 316 (6.05) 215 (4.11) 47 (0.90) 87 (1.66) 9 (0.17) 0 (0)	1273 (27.14) 1313 (27.99) 742 (15.82) 747 (15.92) 166 (3.54) 376 (8.02) 71 (1.51) 3 (0.06)	4546 (45.83) 2593 (26.14) 1058 (10.67) 962 (9.70) 213 (2.15) 463 (4.67) 80 (0.81) 3 (0.03)
**Depression** N (%) **Yes** **No**	218 (4.17) 5009 (95.83)	97 (2.07) 4594 (97.93)	315 (3.18) 9603 (96.82)
**Chronic headaches** N (%) **Yes** **No**	285 (6.08) 4406 (93.92)	787 (15.6) 4440 (84.94)	1072 (10.81) 8846 (89.19)
**BMI** (kg/m²) M (SD)	28.56 (4.88)	26.34 (4.05)	27.51 (4.64)
**Physical activity level** (METs/hours) M (SD)	39.32 (4.52)	42.89 (10.63)	41.01 (8.20)
**Dietary iron intake (**milligrams per day**)** M (SD)	16.53 (6.41)	23.10 (8.38)	19.64 (8.10)

* Me (SD): Mean (Standard Deviation).

**N (%): Number (percent).

Prior to conducting the path analysis, a thorough preliminary examination of the dataset was undertaken to assess normality, identify potential outliers, and evaluate multicollinearity among the variables. No substantial deviations from normality or extreme outlier values were detected. Additionally, Pearson correlation coefficients among the study variables were all below 0.41 (Table 2), and VIF values for the independent variables ranged from 1.00 to 1.08. These results indicate that multicollinearity was not a concern in this dataset.

**Table 2:** Bivariate correlations between studied Variables.

	Age	Gender	Marital Status	Educational Level	Depression	Physical activity	BMI	Dietary iron intake	Chronic Headaches
**Age**	1	0.04^**^	0.18^**^	0.41^**^	0.03^**^	− 0.03^**^	− 0.03^**^	− 0.13^**^	− 0.01
**Gender**	0.04^**^	1	0.08^**^	0.28^**^	0.06^**^	− 0.22^**^	0.24^**^	− 0.40^**^	0.14^**^
**Marital Status**	0.18^**^	0.08^**^	1	0.11^**^	0.01	− 0.03^**^	0.05^**^	− 0.04^**^	0.03^**^
**Educational Level**	0.41^**^	0.28^**^	0.11^**^	1	0.01	0.001	0.05^**^	− 0.13^**^	0.02
**Depression**	0.03^**^	0.06^**^	0.01	0.01	1	− 0.05^**^	0.04^**^	− 0.03^**^	0.07^**^
**Physical activity**	− 0.03^**^	− 0.22^**^	− 0.03^**^	0.001	− 0.05^**^	1	− 0.15^**^	0.22^**^	− 0.03^**^
**BMI**	− 0.03^**^	0.24^**^	0.05^**^	0.05^**^	0.04^**^	− 0.15^**^	1	− 0.01	0.06^**^
**Dietary iron intake**	− 0.13^**^	− 0.40^**^	− 0.04^**^	− 0.13^**^	− 0.03^**^	0.22^**^	− 0.01	1	− 0.03^*^
**Chronic Headaches**	− 0.01	0.14^**^	0.03^**^	0.02	0.07^**^	− 0.03^**^	0.06^**^	− 0.03^*^	1

**The correlation is significant at the 0.01 level.

*The correlation is significant at the 0.05 level.

Table 2 summarizes the bivariate correlations among the key study variables.

The results of the Pearson correlation analysis revealed that age was significantly associated with depression, physical activity, BMI, and dietary iron intake. Similarly, gender demonstrated significant correlations with all of these variables, as well as with chronic headaches. Marital status was significantly correlated with physical activity, BMI, dietary iron intake, and chronic headaches. Additionally, educational level showed significant associations with BMI and dietary iron intake (*P* < 0.01). Based on these findings, age, gender, marital status, and educational level were included as covariates in the mediation models to account for potential confounding effects.

### Path analysis results

A path model was employed to examine the associations between depression and chronic headaches, with physical activity, BMI, and dietary iron intake considered as mediating variables. The model demonstrated an acceptable fit to the data (Table 3). Table 4 presents the path coefficients for the direct effects, including both standardized and unstandardized estimates, along with their corresponding confidence intervals.

**Table 3:** Model fit indices of the estimated Model.

Statistic	Model
χ2	0.39
df	1
CFI	1
TLI	1
SRMR	0.001
RMSEA	0.000
AIC	201103.67
BIC	201341.34

Note: *N* = 9918 participants. χ2: Chi-square test statistic; df: degree of freedom; CFI: comparative fit index; TLI: Tucker-Lewis index; SRMR: standardized root mean square residual; RMSEA: root-mean-square error of approximation; AIC: Akaike’s information criterion; BIC: Bayesian information criterion.

Several significant direct effects were identified in the model. Depression was negatively associated with physical activity (β = −0.04, 95% CI [−0.06, −0.03]) and dietary iron intake (β = −0.03, 95% CI [−0.05, −0.02]), and positively associated with BMI (β = 0.03, 95% CI [0.01, 0.05]). Furthermore, BMI (β = 0.03, 95% CI [0.01, 0.05]) and dietary iron intake (β = −0.05, 95% CI [−0.06, −0.03]) were significantly associated with chronic headaches, whereas the effect of physical activity on chronic headaches was not statistically significant (β = 0.01, 95% CI [−0.01, 0.02]).

Physical activity also showed significant associations with BMI (β = −0.14, 95% CI [−0.15, −0.12]) and dietary iron intake (β = 0.24, 95% CI [−0.22, 0.25]). In addition, a direct positive effect of depression on chronic headaches was observed (β = 0.07, 95% CI [0.05, 0.08]) (Table 4).

Given the significant direct effect of depression on chronic headaches (β = 0.07, 95% CI [0.05, 0.08], *p* < 0.001), along with significant indirect effects through BMI and dietary iron intake, the results support a model of partial mediation rather than full mediation. Although physical activity did not have a direct effect on chronic headaches, it contributed indirectly through its associations with BMI and dietary iron intake. These findings indicate the presence of multiple pathways with partial mediation.

**Fig. 2:**
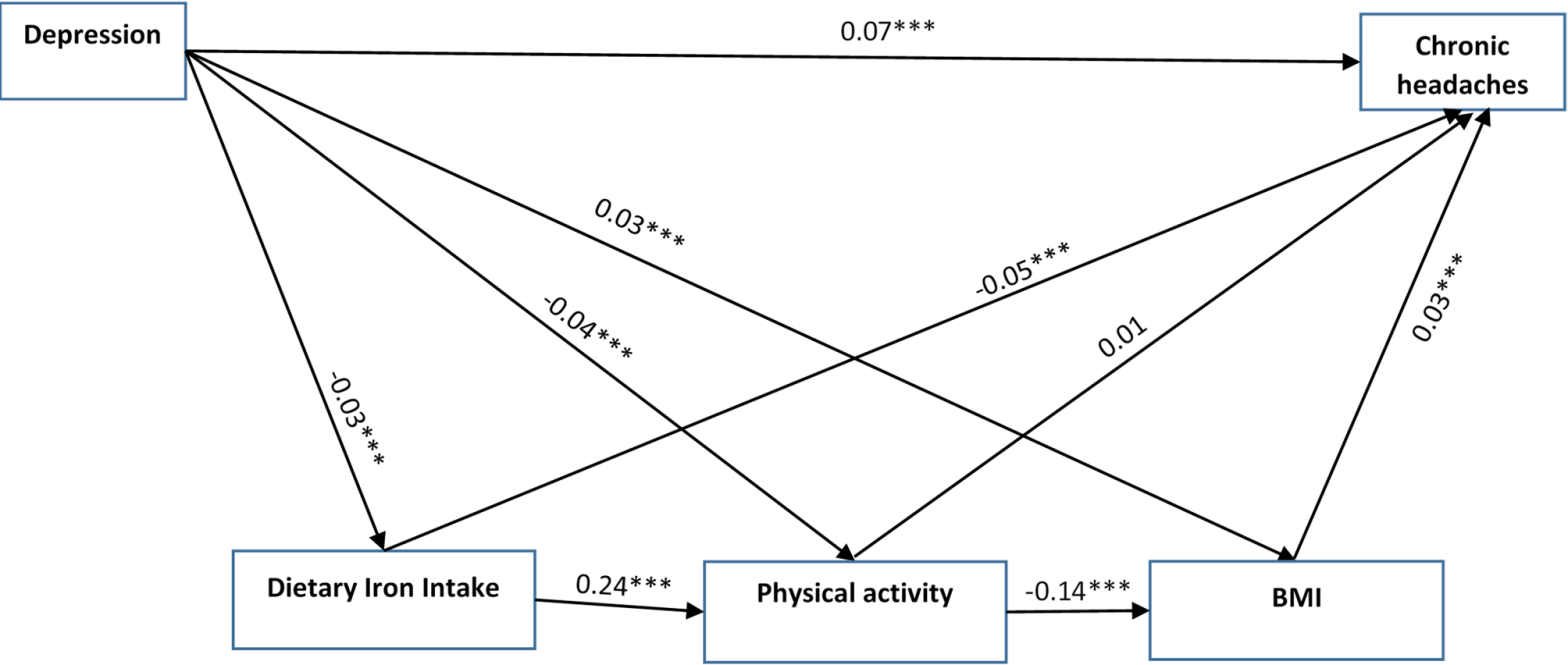
The mediation role of physical activity, body mass index, and dietary iron intake in the relationship between depression and chronic headaches. Age, gender, marital status and educational level were controlled in the model. Note: BMI = Body Mass Index; ***P < 0.001.

The direct path coefficients are summarized in Table 4 and visually represented in Fig. 2.

**Table 4:** Path coefficients of direct effects among variables in the estimated model (*N* = 9918).

Effect	Independent variable	Dependent variable	B	β	SE	95% CI
Direct effect	**Depression**	**Physical activity**	−1.95 ***	−0.04	0.01	[−0.06, −0.03]
		**BMI**	0.83 ***	0.03	0.01	[0.01, 0.05]
		**Dietary iron intake**	−1.48***	−0.03	0.01	[−0.05, −0.02]
	**Physical activity**	**chronic headaches**	0.00	0.01	0.01	[−0.01, 0.02]
	**BMI**	**chronic headaches**	0.002 ***	0.03	0.01	[0.01, 0.05]
	**Dietary iron intake**	**chronic headaches**	−0.002***	−0.05	0.01	[−0.06, −0.03]
	**Physical activity**	**BMI**	−0.08***	−0.14	0.01	[−0.15, −0.12]
	**Dietary iron intake**	**Physical activity**	0.24***	0.24	0.01	[0.22, 0.25]
	**Depression**	**chronic headaches**	0.12***	0.07	0.01	[0.05, 0.08]

Note: B = Unstandardized coefficient; β = Standardized coefficient; CI = 95% Confidence interval; SE = Standard error; BMI = Body Mass Index; ***P ≤ 0.001; **P ≤ 0.01 *P < 0.05. Age, gender, marital status and educational level were controlled in the model.

The results revealed significant indirect effects of depression on chronic headaches through BMI (β = 0.001, 95% CI [0.00, 0.002]) and dietary iron intake (β = 0.002, 95% CI [0.001, 0.002]). In contrast, the indirect pathway through physical activity was not statistically significant (β = 0.00, 95% CI [−0.001, 0.00]) (Table 5).

**Table 5:** Path coefficients of indirect effects among variables in the estimated model (*N* = 9918).

Effect	Independent variable	Mediating variable	Dependent variable	B	β	SE	95% CI
Indirect effect	**Depression**	**Physical activity**	**Chronic headaches**	−0.001	0.00	0.00	[−0.001, 0.00]
		**BMI**		0.002*	0.001	0.00	[0.000, 0.002]
		**Dietary iron intake**		0.003**	0.002	0.001	[0.001, 0.002]
	**Total indirect**			0.004**	0.002	0.001	[0.001, 0.004]
	**Total**			0.12***	0.07	0.01	[0.05, 0.08]

Note: B = Unstandardized coefficient; β = Standardized coefficient; CI = 95% Confidence interval; SE = Standard error; BMI = Body Mass Index; ***P < 0.001; **P ≤ 0.01 *P < 0.05. Age, gender, marital status and educational level were controlled in the model.

The analysis indicates that both BMI and physical activity function as mediators in a serial causal pathway linking depression to chronic headaches. Moreover, the association between depression and chronic headaches was sequentially mediated by physical activity, BMI, and dietary iron intake. Both the direct effect (β = 0.07, 95% CI [0.05, 0.08]) and the total indirect effect (β = 0.002, 95% CI [0.001, 0.004]) of depression on chronic headaches were statistically significant.

## Discussion

Depression is a major psychiatric condition consistently linked to chronic headaches^40^. Understanding the pathways through which depression influences headache outcomes may help guide preventive and therapeutic strategies. This study examined whether physical activity, BMI, and dietary iron intake mediate the association between depression and chronic headaches using path analysis.

The path model demonstrated an acceptable fit and suggested an indirect association between depression and chronic headaches, potentially mediated by BMI and dietary iron intake. The data also showed a significant direct association between depression and chronic headaches. These findings are consistent with previous research, such as studies by Bintari et al. (2021)^2^ and Underwood et al. (2023)^41^, which have reported strong correlations between depression severity and headache frequency or intensity. For example, Bintari et al. (2021) found a statistically significant relationship between higher depression scores and greater headache severity^2^, while Chu et al. (2018)^42^ reported that individuals with depression tended to experience more frequent and intense migraines than non-depressed individuals.

The findings suggest a statistically significant association between depression and chronic headaches, which may involve multiple pathways. Specifically, this study explored the potential mediating roles of physical activity, BMI, and dietary iron intake. Existing literature is consistent with these pathways, noting that neuroinflammation and alterations in pain perception^43,44^ might contribute to the relationship between depression and chronic headaches. These insights may inform future research aimed at understanding the complex interplay of biological, psychological, and social factors involved in both conditions.

Higher levels of depression were associated with lower physical activity. However, physical activity was not significantly related to chronic headache, and the indirect pathway through physical activity did not reach significance.

Previous research has consistently found a negative association between depression and physical activity^14–16^. People with higher depression scores often lead more sedentary lifestyles^45,46^. Wernhart et al^47^. similarly observed that depressive symptoms were associated with increased sedentary behavior. These results match our findings, where higher depression was linked to lower physical activity. Reduced motivation and low energy may help explain this relationship^48^.

Conversely, although some studies have reported an association between physical activity and headache prevalence^17^, this association was not observed in the present study. The discrepancy may reflect differences in study design, methods for assessing physical activity and headache characteristics, or population-specific factors.

In our analysis, physical activity did not mediate the relationship between depression and chronic headaches. This suggests that other factors, possibly biological or behavioral, may play a more prominent role in this association. Future longitudinal and experimental studies are needed to clarify these complex dynamics.

The current study revealed that depression was associated with increased BMI, which was in turn related to greater occurrence of chronic headaches. The mediation analysis suggested a potential indirect pathway through which depressive symptoms may be linked to chronic headaches via BMI. These associations underscore the complex interplay between mental and physical health.

While previous research has identified associations between depression, BMI, and chronic headaches, the specific mediating role of BMI remains insufficiently examined. Previous studies have reported associations between depressive symptoms and alterations in body composition, including increased visceral fat and central obesity^21,49^. Similarly, higher BMI has been statistically associated with greater prevalence of chronic headaches in various populations^23,24^. Several studies have explored mechanisms underlying these associations. One possibility is that adipose tissue acts as an active endocrine organ. It releases pro-inflammatory cytokines and adipokines, which may affect pain pathways and increase susceptibility to headaches^50,51^. Hormonal changes, particularly in the serotonin and orexin systems, might also play a role. These alterations could influence appetite regulation and neural excitability, factors that are relevant to migraine development^51^. In addition, both physiological and behavioral pathways have been suggested. For instance, some evidence indicates that elevated cortisol levels in individuals with depressive symptoms may be associated with increased fat accumulation and systemic inflammation, which in turn might be linked to metabolic dysregulation^52,53^. Behavioral factors may also be important. Individuals with depressive symptoms often have lower physical activity and poorer dietary habits. These behaviors can increase BMI, which may in turn raise the risk of chronic headaches^54^.

This study highlights a potential mediating role of BMI in the association between depression and chronic headaches. Higher BMI, which may be influenced by depression-related factors such as hormonal imbalance and inflammation, could increase susceptibility to headaches. Adipose tissue acts as an endocrine organ, releasing pro-inflammatory cytokines and adipokines that may affect pain perception. In addition, changes in serotonin and orexin systems associated with depression could alter neural excitability, contributing to headache disorders. These findings emphasize the need for further research to clarify BMI’s role in this complex relationship.

The current study found a statistically significant association between depression and reduced dietary iron intake, and between lower dietary iron intake and greater chronic headache symptoms. Mediation analysis suggested a possible indirect pathway linking depression to chronic headaches through dietary iron intake.

To date, no mediation studies have directly examined the pathway linking depression to chronic headaches via dietary iron intake. However, several mechanisms have been proposed to explain how depression may influence iron consumption. Evidence suggests that depression can disrupt iron metabolism, potentially reducing both absorption and storage, which may contribute to chronic headache susceptibility^4,27,55,56^. Depression has also been associated with changes in hormone levels, including reductions in serotonin and dopamine, which are thought to play a regulatory role in iron metabolism by influencing gene expression related to iron transport and storage^57,58^. In their 2019 review titled *Nutritional Aspects of Depression*, Preeti et al. noted that depression may be linked with decreased appetite and altered eating behaviors, potentially resulting in lower iron intake^59^. Additionally, Lee et al. (2020) discussed that increased physiological demands during depression—such as those stemming from inflammation, stress response, and gut microbiome alterations—might be related to diminished iron absorption and storage^60^.

On the other hand, several studies have examined the relationship between dietary iron intake and chronic headaches^4,11,12^. Meng et al. (2021) analyzed 7,880 adults from the NHANES dataset (1999–2004) and found that higher dietary iron intake was associated with fewer severe headaches or migraines, especially among women aged 20–50^11^. Insufficient consumption of dietary iron has long been recognized as a major contributing factor to the development of iron deficiency, which, if prolonged, may progress to iron deficiency anemia^61^. Iron deficiency has also been associated with headache symptoms in prior studies^62^. Iron deficiency can impair oxygen transport and lead to cerebral hypoxia^63^, which may activate the trigeminovascular system, a key pathway in headache generation^64^. Additionally, iron is essential for synthesizing neurotransmitters such as serotonin and dopamine, which regulate mood and pain perception. Disruptions in these systems may lower the threshold for headache onset^27,57,65^. Iron deficiency is also linked to chronic inflammation and elevated pro-inflammatory cytokines^4,66^, which can contribute to central sensitization and pain amplification^67^. It should be noted that this study assessed dietary iron intake rather than actual iron status. While low intake may reflect a risk of iron deficiency, it does not confirm physiological iron depletion. Therefore, the proposed mechanisms involving impaired oxygen delivery or neurotransmitter dysfunction should be interpreted cautiously, and future studies should include biochemical assessments of iron status to validate this pathway.

Overall, these findings underscore the mediating role of dietary iron intake in the relationship between depression and chronic headaches. Addressing dietary iron deficiency may offer a potential avenue for alleviating headache symptoms in individuals with depression.

The analysis indicated that both BMI and physical activity may function as mediators in a serial manner in the association between depression and chronic headaches. In addition, the relationship between depression and chronic headaches appeared to be sequentially mediated by physical activity, BMI, and dietary iron intake.

In the first sequential model, depression was linked to lower physical activity levels, which were in turn associated with higher BMI and finally a greater likelihood of chronic headaches. Depression is often accompanied by reduced motivation and energy, which can lead to more sedentary behavior. Decreased physical activity may contribute to weight gain through lower energy expenditure and metabolic changes. Elevated BMI, in turn, has been associated with greater frequency or severity of chronic headaches, potentially through mechanisms involving hormonal disturbances, pro-inflammatory cytokine activity, and increased neural excitability related to adipose tissue. In the second model, dietary iron intake appeared to play an additional role. Depression was associated with lower iron consumption, which may influence physical activity, BMI, and ultimately the occurrence of chronic headaches. A similar pattern was reported in the study by Tian et al. (2023), which used cross-sectional data from the NHANES. That study found a significant correlation between higher dietary iron intake and lower depressive symptoms. Moreover, it was reported that BMI may moderate the association between iron intake and depression^68^.

It appears that depression may be associated with altered appetite and eating behaviors, which in turn are related to suboptimal dietary choices, including lower iron intake. Insufficient iron levels have been linked to fatigue and reduced physical performance, which may correspond with lower levels of physical activity. This reduction in activity is often associated with weight gain and a subsequent increase in BMI, which in turn has been linked to greater severity of chronic headaches. Together, these models highlight the complex and multifactorial associations between depression and chronic headaches, involving both nutritional and physical factors.

These results align with the biopsychosocial model of chronic pain, indicating that depression (a psychological factor) may contribute to chronic headaches (a biological outcome) through mediators such as physical activity, BMI, and dietary iron intake. These mediators may represent biological and behavioral pathways linking depression with chronic pain. The results emphasize that mental and physical health are closely intertwined, pointing to the value of more integrated approaches in managing chronic headaches. Clinically, addressing both emotional symptoms and lifestyle factors—such as promoting regular physical activity, improving nutrition (particularly iron intake), and maintaining a healthy weight—could help reduce headache burden in individuals with depression. Holistic programs that combine mental-health support with lifestyle interventions may further prevent or lessen the co-occurrence of depression and chronic pain.

### Limitations

This study has several limitations that should be considered when interpreting the findings. The cross-sectional design inherently restricts causal inferences regarding the relationships among depression, chronic headaches, and the proposed mediators. Prospective longitudinal studies are needed to elucidate the temporal ordering and directionality of these associations. Measurement limitations arise from the reliance on self-reported data for physical activity and dietary iron intake (assessed via FFQ), which may be susceptible to recall bias and misreporting, potentially impacting the accuracy of the estimated pathways. Furthermore, dietary iron intake was used solely as an indirect proxy for iron status; biochemical markers such as serum ferritin or hemoglobin were not available, limiting the biological validation of this mediator. The absence of headache subtype classification (e.g., migraine versus tension-type headache) constitutes another important limitation, as these conditions may differentially interact with depression and mediating factors, thereby influencing path coefficients and model fit. Selection bias is also a potential concern, as the cohort characteristics may limit the generalizability of the results to more heterogeneous populations. In addition, depression was operationalized using a binary measure, which does not capture the full dimensional spectrum of depressive symptomatology, possibly attenuating the sensitivity of the modeled relationships. Lastly, unmeasured confounding variables, including medication use and comorbid health conditions, were not fully controlled for, which may have introduced residual confounding in the structural pathways examined. Future research should address these methodological constraints by incorporating longitudinal designs, objective biomarkers, detailed headache phenotyping, and more nuanced measures of depression to enhance the validity and applicability of path analytic models in this field.

## Conclusion

This study contributes to the understanding of the complex associations between depression and chronic headaches by identifying the potential mediating roles of physical activity, BMI, and dietary iron intake, situated within the biopsychosocial framework. The results suggest that depressive symptoms are associated with chronic headaches through interconnected behavioral and physiological factors. These findings underscore the importance of adopting an integrative approach that considers psychological, biological, and lifestyle components in the context of chronic headache management. Future research employing longitudinal designs and more heterogeneous samples may help to further elucidate these pathways and improve the generalizability of findings. Such investigations could guide the development of multidimensional strategies to manage chronic headaches and enhance overall well-being.

## Supplementary Information

Below is the link to the electronic supplementary material.

Supplementary Material 1

## Data Availability

The datasets used and/or analyzed during the current study are available from the corresponding author on reasonable request.

## Abbreviations

BMI: Body mass index
RaNCD: Ravansar non-communicable disease
FFQ: Food frequency questionnaire
PERSIAN: Prospective epidemiological Re-search in IrAN
MET: Metabolic equivalent of task
SPSS: Statistical package for social sciences
STROBE: Strengthening the reporting of observational studies in epidemiology
VIF: Variance inflation factor
SFAs: Saturated fats
MUFA: Monounsaturated fatty acids
PUFA: Polyunsaturated fatty acids
EFA: Explanatory factor analysis
CFA: Confirmatory factor analysis
KMO: Kaiser-meyer-olkin index
AVE: Average variance extracted
CR: Composite reliability
WLSMV: Weighted least squares
TLI: Tucker-lewis index
CFI: Comparative fit index
RMSEA: Root mean square error of approximation
SRMR: Standardized root mean square residual
NHANES: National health and nutrition examination survey
