# Supplementary material for: Mediating effects of physical activity, BMI, and dietary iron intake on the relationship between depression and chronic headaches
Source: Sci Rep. 2025 Dec 18;16:2216. doi: 10.1038/s41598-025-31993-0 (PMC12816051; doi:10.1038/s41598-025-31993-0)
Supplement: Supplementary file 1 — Supplementary Material 1 [file 41598_2025_31993_MOESM1_ESM.docx]

**Supplementary File**

**STROBE Statement—Checklist with Section and Page References**

**Title and Abstract**

**Item 1(a): Indicate the study’s design with a commonly used term in the title or the abstract**

- **Section**: Abstract
- **Page**: 2
- **Details**: The abstract explicitly states “cross-sectional approach” and mentions “path analysis,” clearly indicating the study design.

**Item 1(b): Provide in the abstract an informative and balanced summary of what was done and what was found**

- **Section**: Abstract
- **Page**: 2
- **Details**: The abstract summarizes the methods (cross-sectional study, 9918 participants, path analysis, mediators) and findings (direct effect β=0.07, indirect effects via BMI and dietary iron intake, non-significant via physical activity).

**Introduction**

**Item 2: Explain the scientific background and rationale for the investigation being reported**

- **Section**: Introduction
- **Pages**: 3–5
- **Details**: The introduction discusses depression, chronic headaches, the biopsychosocial model, and literature gaps, providing the rationale for studying mediators (physical activity, BMI, dietary iron intake).

**Item 3: State specific objectives, including any prespecified hypotheses**

- **Section**: Introduction
- **Page**: 5
- **Details**: The objective is stated as investigating the mediating role of physical activity, BMI, and dietary iron intake in the depression–chronic headaches relationship, with a hypothesis of significant joint mediation.

**Methods**

**Item 4: Present key elements of study design early in the paper**

- **Section**: Material and Methods (Study Design and Participants)
- **Page**: 5-6
- **Details**: The section describes the cross-sectional mediation analysis using RaNCD cohort data, specifying the population (ages 35–65) and path analysis approach.

**Item 5: Describe the setting, locations, and relevant dates, including periods of recruitment, exposure, follow-up, and data collection**

- **Section**: Material and Methods (Study Design and Participants)
- **Page**: 5-8
- **Details**: The setting is Ravansar, Kermanshah, Iran, with data from the RaNCD cohort’s recruitment phase (starting November 2014).

**Item 6(a): Give the eligibility criteria, and the sources and methods of selection of participants**

- **Section**: Material and Methods (Study Design and Participants)
- **Page**: 5-6
- **Details**: Eligibility criteria include ages 35–65 from the RaNCD cohort; exclusion criteria cover unwillingness, short residency, disabilities, and missing data (n=130). Selection is via available sampling.

**Item 7: Clearly define all outcomes, exposures, predictors, potential confounders, and effect modifiers. Give diagnostic criteria, if applicable**

- **Section**: Material and Methods (Data Collection)
- **Pages**: 5–11
- **Details**: Defines chronic headaches (outcome, ≥15 days/month for ≥3 months), depression (exposure, psychologist assessment/self-reported medication), mediators (physical activity, BMI, dietary iron intake), and confounders (age, gender, marital status, educational level).

**Item 8: For each variable of interest, give sources of data and details of methods of assessment (measurement). Describe comparability of assessment methods if there is more than one group**

- **Section**: Material and Methods (Data Collection)
- **Pages**: 5–11
- **Details**: Specifies data sources: digital questionnaires (sociodemographic, depression), PERSIAN cohort questionnaire (physical activity), Inbody 770 (BMI), FFQ (dietary iron), and standard criteria (chronic headaches). No group comparisons are noted.

**Item 9: Describe any efforts to address potential sources of bias**

- **Section**: Material and Methods (Data Collection, Statistical Analyses)
- **Pages**: 6–11
- **Details**: Mentions rigorous data collection (interviews, supervisor scrutiny) and statistical checks (normality, outliers, multicollinearity, VIF 1–1.08). Self-report/selection bias is not explicitly addressed.

**Item 10: Explain how the study size was arrived at**

- **Section**: Material and Methods (Study Design and Participants)
- **Page**: 6-9
- **Details**: Reports 9,918 participants after excluding 130

The study used data from the RaNCD cohort, which included 10,048 participants. For the present analysis, we included 9,918 individuals based on the availability of complete data on the variables of interest (depression, physical activity, BMI, Dietary iron intake and chronic headache). No formal sample size calculation was performed, as this is a secondary data analysis using an existing cohort dataset.

**Item 11: Explain how quantitative variables were handled in the analyses. If applicable, describe which groupings were chosen and why**

- **Section**: Material and Methods (Data Collection, Statistical Analyses)
- **Pages**: 8–11
- **Details**: Physical activity is categorized (low, moderate, high METs-hours) with PERSIAN cohort cutoffs; BMI and dietary iron intake are continuous. Handling is described in path analysis.

**Item 12(a): Describe all statistical methods, including those used to control for confounding**

- **Section**: Material and Methods (Statistical Analyses)
- **Pages**: 10–11
- **Details**: Describes descriptive statistics, normality/outlier checks, path analysis (maximum likelihood, 5,000 bootstraps), and control for confounders (age, gender, marital status, educational level).

**Item 12(b): Describe any methods used to examine subgroups and interactions**

- **Section**: Not Applicable
- **Page**: N/A
- **Details**: No subgroup or interaction analyses are reported, as the study focuses on mediation across the entire sample.

**Item 12(c): Explain how missing data were addressed**

- **Section**: Material and Methods (Statistical Analyses)
- **Page**: 10
- **Details**: Reports 130 cases (1.3%) with missing data excluded via listwise deletion, justified by minimal impact.

**Item 12(d): If applicable, describe analytical methods taking account of sampling strategy**

- **Section**: Material and Methods (Study Design and Participants)
- **Page**: 7
- **Details**: Mentions available sampling but does not describe specific analytical methods (e.g., weighting) to account for it.

As this was a secondary data analysis of the RaNCD cohort study and no complex sampling design adjustments were applied in the current analysis, analytical methods did not account for sampling strategy.

**Item 12(e): Describe any sensitivity analyses**

- **Section**: Material and Methods (Statistical Analyses)
- **Page**: 11
- **Details**:

To assess the robustness of the model, sensitivity analyses were conducted. In this regard, all potential confounding variables—such as age, gender, education level, marital status, and socioeconomic status—were statistically controlled for across all tested pathways. Second, alternative model specifications were tested by examining the effects of each mediator (e.g., physical activity, BMI, and dietary iron intake) separately and in combination. The results remained consistent in direction and significance across models, suggesting that the associations identified in the main model are stable and not highly sensitive to alternative model structures or omitted confounders.

**Results**

**Item 13(a): Report numbers of individuals at each stage of study—e.g., numbers potentially eligible, examined for eligibility, confirmed eligible, included in the study, completing follow-up, and analysed**

- **Section**: Material and Methods (Study Design and Participants), Results
- **Pages**: 7, 12
- **Details**: Reports 10,048 initial participants, 130 excluded, and 9,918 analyzed (Page 7). Results confirm 9,918 participants (Page 12).

**Item 13(b): Give reasons for non-participation at each stage**

- **Section**: Material and Methods (Study Design and Participants)
- **Page**: 7
- **Details**: missing data (n=130).

**Item 13(c): Consider use of a flow diagram**

- **Section**: Material and Methods
- **Page**: 10
- **Details**: References a STROBE flow chart (Figure 1) showing participant selection stages.

**Item 14(a): Give characteristics of study participants (e.g., demographic, clinical, social) and information on exposures and potential confounders**

- **Section**: Results (Table 1)
- **Page**: 12
- **Details**: Table 1 details age, gender, marital status, education, depression, chronic headaches, BMI, physical activity, and dietary iron intake.

**Item 14(b): Indicate number of participants with missing data for each variable of interest**

- **Section**: Material and Methods (Statistical Analyses)
- **Page**: 10
- **Details**: Of the initial 10,048 participants, 130 cases (1.3%) with missing data on the primary study variables were excluded from the analysis. The number of missing cases per variable was as follows: depression (n = 42), physical activity (n = 35), BMI (n = 18), dietary iron intake (n = 25), and headache frequency (n = 10).

**Item 15: Report numbers of outcome events or summary measures**

- **Section**: Results (Table 1)
- **Page**: 12
- **Details**: Reports chronic headaches (10.81%, n=1072) and no headaches (89.19%, n=8846), with summary measures for other variables.

**Item 16(a): Give unadjusted estimates and, if applicable, confounder-adjusted estimates and their precision (e.g., 95% confidence interval). Make clear which confounders were adjusted for and why they were included**

- **Section**: Results (Tables 4, 5)
- **Pages**: 16–17
- **Details**: Provides confounder-adjusted estimates (e.g., β=0.07, 95% CI: 0.05–0.08 for depression → chronic headaches) with confounders (age, gender, marital status, educational level) justified via correlations (Table 2, Page 14).

**Item 16(b): Report category boundaries when continuous variables were categorized**

- **Section**: Material and Methods (Data Collection)
- **Page**: 8
- **Details**: Physical activity categories (low: 24–36.5 METs-hours, moderate: 36.6–44.9 METs-hours, high: ≥45 METs-hours) are reported.

**Item 16(c): If relevant, consider translating estimates of relative risk into absolute risk for a meaningful time period**

- **Section**: Not Applicable
- **Page**: N/A
- **Details**: Path analysis uses standardized coefficients (β), not relative risks, so this item is not applicable.

**Item 17: Report other analyses done—e.g., analyses of subgroups and interactions, and sensitivity analyses**

- **Section**: Material and Methods (Statistical Analyses), Results
- **Pages**: 11, 16–17
- **Details**: Sensitivity analyses (Page 11) test the direct effect (β=0.117), with results referenced in Tables 4 and 5 (Pages 16–17). No subgroup/interaction analyses are reported.

**Discussion**

**Item 18: Summarise key results with reference to study objectives**

- **Section**: Discussion
- **Page**: 18
- **Details**: Summarizes direct (β=0.07) and indirect effects (via BMI, dietary iron intake) in relation to the mediation objective.

**Item 19: Discuss limitations of the study, taking into account sources of potential bias or imprecision. Discuss both direction and magnitude of any potential bias**

- **Section**: Discussion (Limitations)
- **Page**: 22
- **Details**: Mentions cross-sectional design, self-report bias, and generalizability but does not fully discuss bias direction/magnitude.

**Item 20: Give a cautious overall interpretation of results considering objectives, limitations, multiplicity of analyses, results from similar studies, and other relevant evidence**

- **Section**: Discussion
- **Pages**: 18–22
- **Details**: Interprets results cautiously, linking to the biopsychosocial model, comparing with prior studies, and noting limitations.

**Item 21: Discuss the generalisability (external validity) of the study results**

- **Section**: Discussion (Limitations)
- **Page**: 22
- **Details**: Notes that the Kurdish sample may limit generalizability to other populations.

**Other Information**

**Item 22: Give the source of funding and the role of the funders for the present study and, if applicable, for the original study on which the present article is based**

- **Section**: Declarations (Funding/Support)
- **Page**: 23
- **Details**: Lists funding from Kermanshah University of Medical Sciences (grant 92472) and the Iranian Ministry of Health (Grant 700/534).
